# Targeting G9a Exerts Pleiotropic Suppression in Triple-Negative Breast Cancer Cells: Cooperatively Inducing Pyroptosis and Apoptosis

**DOI:** 10.3390/biom16030345

**Published:** 2026-02-25

**Authors:** Jialin Li, Guijuan Zhang, Tianyang Liu, Xianxin Yan, Min Ma

**Affiliations:** 1 The First School of Clinical Medicine, State Key Laboratory of Bioactive Molecules and Druggability Assessment, Guangdong Basic Research Center of Excellence for Natural Bioactive Molecules and Discovery of Innovative Drugs, Jinan University, Guangzhou 510632, China; 2 School of Nursing, Jinan University, Guangzhou 510632, China; 3 School of Traditional Chinese Medicine, Jinan University, No. 601, West Huangpu Avenue, Guangzhou 510632, China

**Keywords:** triple-negative breast cancer, pyroptosis, G9a, BIX-01294, RIG-I/STAT1/GSDME

## Abstract

**Background:** Pyroptosis, a pro-inflammatory programmed cell death process, is a key player in tumor biology, including in triple-negative breast cancer (TNBC). Inhibiting G9a has been proven to exert anticancer effects; however, the molecular mechanism of the effects remains unclear. The study aimed to illustrate whether inhibiting G9a can suppress the process of TNBC cells by promoting pyroptosis and investigate the underlying mechanisms. **Methods:** MCF-10A, MDA-MB-231 and SUM159PT cell lines were used for in vitro study. CCK8 and EdU staining assay were used to examine the cell proliferation, and flow cytometry assay was performed to evaluate cell death. Inflammatory factors were measured by ELISA kits. The mRNA and protein expression levels were analyzed by qRT-PCR, Western blot, and immunofluorescence staining. Transmission electron microscopy was used to observe the morphological changes in cells. **Results:** We found that knockdown of G9a suppressed the growth and the abilities of invasion and migration, induced pyroptosis, and increased the expression of RIG-I, p-STAT1, and GSDME of TNBC. Furthermore, a RIG-I inhibition Cyclo (Phe-Pro) partially rescued the activation of pyroptosis enhanced by knockdown of G9a. **Conclusions:** These findings indicate that inhibiting the function of G9a induces pyroptosis in TNBC cells by the RIG-1/STAT1/GSDME pathway, which provides a new therapeutic target for TNBC treatment.

## 1. Introduction

Breast cancer (BC) is the leading cancer diagnosis among women and still has a high mortality rate in developing and low-income countries [1]. According to three molecular markers, which are known as estrogen receptor (ER), progesterone receptor (PR), as well as human epidermal growth factor receptor 2 (HER2), BC is classified into four types: luminal A, luminal B, HER2-positive, and triple-negative breast cancer (TNBC) [2]. Among them, TNBC is further categorized into six subtypes, which are Basal-Like 1 (BL1), Basal-Like 2 (BL2), Immunomodulatory (IM), Mesenchymal (M), Mesenchymal Stem-Like (MSL), and Luminal Androgen Receptor (LAR) [3]. TNBC accounts for approximately 10–20% of all types but represents an aggressive subtype associated with poor prognosis [1]. Although the therapeutic impact of BC has increased with ongoing treatment updates, patients with TNBC may not respond effectively because of the lack of receptor indicators. For TNBC, there is still a lack of effective therapy so far, and chemotherapy is still the top choice for patients who lost the opportunity for surgery [4]. The prognosis is much worse than that of the other subtypes since fewer patients with TNBC could achieve a full response due to its high invasiveness and recurrence [5,6]. Therefore, seeking safe and efficient methods and therapeutic targets is of great significance for patients with TNBC.

Pyroptosis, a recently identified novel form of programmed cell death mechanism, is related to multiple diseases and plays a significant role in tumors [7,8]. Pyroptosis is executed when the Gasdermin N-terminal forms plasma membrane pores. This results in the massive release of intracellular contents and a potent inflammatory immune response, culminating in this lytic form of cell death [9]. As a member of the Gasdermin family, GSDME is cleaved by Caspase-3. The resulting N-terminal fragment (GSDME-N) then induces pyroptosis [10]. Numerous studies on TNBC indicated that inducing pyroptosis could enhance the efficacy of chemotherapy and radiotherapy, thereby promoting tumor cell death [11,12,13,14].

G9a, also known as EHMT2, is a histone 3 lysine 9 (H3K9) methyltransferase that methylates histone 3 lysine 9 to H3K9me1 and H3K9me2 [15]. It has been discovered that G9a can repress tumor suppressor genes through H3K9 mono-/di-methylation and promote the oncogenes’ functions [16]. Previous studies indicated the role of G9a in tumorigenesis, and G9a is overexpressed in a variety of tumor types, including BC [17,18,19]. In TNBC, G9a knockdown reduces tumor growth and metastasis, suggesting that G9a may be a viable therapeutic target due to its deregulated function [20,21,22]. However, evidence for the role of G9a in TNBC pyroptosis remains scarce, and the underlying molecular mechanisms are poorly understood.

Retinoic acid-induced gene 1 (RIG-I), also known as DDX58, was first identified in 1997 during studies of trans-retinoic acid-induced differentiation in acute promyelocytic leukemia cell lines [23]. Previous studies have shown that RIG-I can regulate the expression of inflammasomes related to pyroptosis [24,25,26] and also takes a great part in cancers [27,28,29,30,31]. Evidence from glioma research suggests a regulatory role for G9a in controlling RIG-I [32].

In this study, we investigated the anti-cancer effects of G9a in TNBC cells, focusing on its role in pyroptosis. We identified the RIG-I/STAT1/GSDME pathway as a potential mechanism through which G9a knockdown induces pyroptosis. These findings position G9a as a promising therapeutic target and offer a novel treatment perspective for TNBC.

## 2. Materials and Methods

### 2.1. Cell Culture

MCF-10A, MDA-MB-231, and SUM159PT were purchased from the American Type Culture Collection (ATCC, Manassas, VA, USA). MCF-10A was cultured in its specific medium (Procell, Wuhan, China). MDA-MB-231 and SUM159PT were cultured in DMEM medium (Gibco, Grand Island, NY, USA) containing 10% fetal bovine serum (Procell, Wuhan, China) and 1% penicillin-streptomycin (Gibco, Grand Island, NY, USA). All cells were incubated at 37 °C with 5% CO_2_.

### 2.2. Reagents

BIX-01294 (HY-108239), Z-VAD-FMK (HY-16658B, purity ≥ 98.78%), and Cyclo (Phe-Pro) (HY-P1934, purity ≥ 98.77%) were purchased from MedChemExpress (MCE, Monmouth Junction, NJ, USA).

### 2.3. Differential Expression Levels of G9a

The UALCAN database was used to determine the differential expression of G9a in normal and tumor tissues from the TCGA database.

### 2.4. RNA Interference

The siRNAs used to carry out the G9a knockdown were synthesized by Sangon Biotech (Shanghai, China). Lipofectamine RNAiMAX (Invitrogen, Carlsbad, CA, USA) was used for the transfection following the manufacturer’s instruction. The sequences were as follows: siG9a #1: 5′-AGAAGAAUUCAAGAGAUUCCG-3′; siG9a #2: 5′-AGUAACGGGCAUCAAUGC-3′; siG9a #3: 5′-CCAUGAACAUCGAUCGCAATT-3′.

### 2.5. Cell Viability Assay

Cells were treated with BIX-01294 (0, 5, 10, 20, and 40 μmol/L) for 24 h, 48 h, and 72 h. Then a Cell Counting Kit-8 (CCK-8) solution (Beyotime, Shanghai, China) was added to each well for another 1 h incubation. The absorbance was measured by a microplate reader (Agilent, Santa Clara, CA, USA) at 450 nm.

### 2.6. EdU Staining

EdU Staining Kit (C0071S, Beyotime, Shanghai, China) was used to measure the EdU in different treatment groups. Cells were seeded into 96-well plates and treated with different treatments. Subsequently, a 2× EdU working solution was prepared according to the manufacturer’s instructions. Then the working solution was added to each well, followed by an additional incubation for 2 h. After removing the medium, the cells were fixed with 4% paraformaldehyde at room temperature for 15 min. PBS with 0.3% Triton X-100 was then added after the fixation and the mixture was incubated at room temperature for 15 min. After that, a click-reaction solution was added to the washed plates, followed by incubation at room temperature in the dark for 30 min. Finally, Hoechst staining solution was added for nuclear staining for 10 min. A fluorescence microscope (ZEISS, Oberkochen, Germany) was used for observation.

### 2.7. Flow Cytometric Assay

Adherent cells were collected using trypsin without EDTA (Biosharp, Shanghai, China), washed with PBS, and centrifuged at 2000 rpm for 5 min twice. Then the cells were re-suspended in binding buffer and stained with Annexin V-FITC and PI (Dojindo, Kumamoto, Japan) for 15 min at room temperature in the dark. Then the cells were analyzed with a flow cytometer (NovoCyte, Agilent, CA, USA).

### 2.8. Transwell Invasion Assay

Cells were re-suspended in medium without FBS and then seeded into 24-well plates. 50 μL of Matrigel (Corning, Corning, NY, USA) mixed with serum-free medium (1:8), and 800 μL of medium with 20%FBS were coated into the upper and lower chamber, respectively. Then 400 μL medium without FBS containing 1 × 10^5^ cells was added into the upper chamber. After incubation for 48 h, each insert was fixed and stained with 4% paraformaldehyde and 0.1% crystal violet. A light microscope (ZEISS, Oberkochen, Germany) was used for the observation after the inserts were completely dried.

### 2.9. Wound-Healing Assay

Once cells reached confluence in 6-well plates, scratches were created by a sterile 200 µL pipette tip. Then the cells were incubated for 24 h at 37 °C with serum-free medium. An inverted microscope (ZEISS, Oberkochen, Germany) was used to detect the width of the scratch, which was measured at 0 h and 24 h.

### 2.10. ELISA and LDH Release Assay

The levels of IL-1β and IL-18 were measured by using a Human IL-1β ELISA Kit (JL13662, Jianglai Biotechnology, Shanghai, China) and a Human IL-18 ELISA Kit (JL19261, Jianglai Biotechnology, Shanghai, China). Cell supernatants from different groups were added to a 96-well plate. After applying a plate sealer, the plate was incubated at 37 °C for 60 min. A biotin-antibody solution was added to each well after the liquid was discarded, and the plate was incubated at 37 °C for an additional 60 min. After the incubation, the plate was washed three times, and streptavidin-HRP was added, followed by incubation at 37 °C for 30 min. After that, the plate was washed five times, and the TMB substrate was added. It was then incubated at 37 °C in the dark for 15 min. After adding stop solution to each well, the optical density (OD) was measured at 450 nm through a microplate reader (Agilent, CA, USA).

LDH levels were detected by a LDH Release Assay Kit (C0019S, Beyotime, Shanghai, China). After adding cell supernatants from different treatment groups to a 96-well plate, LDH release assay buffer was added to adjust the total volume in each well to 100 µL. The standard and sample wells were then filled with 100 µL of the LDH assay working solution and incubated at 37 °C in the dark for 30 min. After the incubation, 20 µL of stop solution was added to each well, and the absorbance was measured at 450 nm.

### 2.11. Transmission Electron Microscopy (TEM)

After treating the cells with different intervention factors, 2.5% glutaraldehyde (G1102, Servicebio, Beijing, China) was used for primary fixation overnight at 4 °C, and 1% osmium tetroxide (02602-AB, Ted Pella, Redding, CA, USA) was used for post-fixation for 2 h at 4 °C in the dark. Subsequently, gradient dehydration was carried out, with each step lasting over 20 min. To enable the resin to completely replace the dehydrating agent and penetrate the cells, the dehydrated samples were placed in a mixture of resin and dehydrating agent before being penetrated in pure resin overnight. The samples were then placed into embedding molds filled with fresh resin and polymerized in an oven at 60 to 70 °C for 48 h to form hard resin blocks. Following this, sectioning (UC-7, Lecia, Wetzlar, Germany) and staining were performed. Finally, the samples were observed at transmission electron microscope (H-7500, HITACHI, Tokyo, Japan).

### 2.12. RT-qPCR Analysis

Cells were seeded in 6-well plates with different treatments. RNA extraction and complementary DNA (cDNA) synthesis were performed according to the manufacturer’s instructions. RNA extraction kit (TCH020, Takara, Kyoto, Japan) and RT Reagent Kit (RR092A, Takara, Kyoto, Japan) were used to extract RNA from the cells and reverse-transcribed into cDNA. TB Green FAST qPCR (RR820A, Takara, Kyoto, Japan) was adopted to detect the quantitative PCR from the 2^−ΔΔCt^ method. The primers were as follows: AIM2: forward 5′-TTGAGAAGAAGGCAAGCCCA-3′, reverse 5′-CGTGAGTATTTACCTCGCGC-3′; Caspase-3: forward 5′-GGACCAAACCTGAAATGTGG-3′, reverse 5′-GACCCTTTGAATTCTGTCCC-3′; GSDME: forward 5′-GGAGGTGCTGGAAGATAGAA-3′, reverse 5′-GTGCGTTGGAGTCCACATTG-3′; IL-1β: forward 5′-CCAGGGACAGGATATGGAGCA-3′; reverse 5′-TTCAACACGCAGGACAGGTACAG-3′; IL-18: forward 5′-CTGCCACCTGCTGCAGTCTA-3′; reverse 5′-TCTACTGGTTCAGCAGCCATCTTTA-3′; β-actin: forward 5′-AGCGAGCATCCCCCAAAGTT-3′; reverse 5′-GGGCACGAAGGCTCATCATT-3′.

### 2.13. Western Blot Assay

Cells were lysed with RIPA Lysis Buffer (Epizyme, Shanghai, China). And BCA assay (Epizyme, Shanghai, China) was used to detect protein concentration. Then 6–10% SDS-PAGE (Epizyme, Shanghai, China) was used to separate proteins depending on the protein molecular weight. After transferring to PVDF membranes (Millipore, Billerica, MA, USA), the membranes were blocked with 5% skim milk to make the primary antibody a better bind. After incubating with primary antibodies at 4 °C overnight, the membranes were then incubated with secondary antibodies for 1 h at room temperature. ECL solution (Biosharp, Shanghai, China) and ChemiDoc MP (Bio-Rad, Hercules, CA, USA) were used to visualize the membranes. The primary antibodies were as follows: anti-G9a (1:5000, #66689-1-Ig, Proteintech, Wuhan, China), anti-H3K9me2 (1:5000, #39041, Active Motif, Shanghai, China), anti-BAX (1:2000, #T40051, Abmart, Shanghai, China), anti-Bcl-2 (1:2000, #T40056, Abmart, Shanghai, China), anti-AIM2 (1:5000, #20590-1-AP, Proteintech, Wuhan, China), anti-cleaved caspase-3 (1:1000, #9664S, CST, Danvers, MA, USA), anti-GSDME (1:5000, #13075-1-AP, Proteintech, Wuhan, China), anti-IL-1β (1:1000, #PC0812, Abmart, Shanghai, China), anti-IL-18 (1:1000, #M027287, Abmart, Shanghai, China), anti-RIG-I (1:5000, #25068-1-AP, Proteintech, Wuhan, China), anti-STAT1 (1:5000, #10144-2-AP, Proteintech, Wuhan, China), anti-p-STAT1 (1:1000, #28977-1-AP, Proteintech, Wuhan, China), anti-β-actin (1:40,000, #66009-1-Ig, Proteintech, Wuhan, China). The secondary antibodies were as follows: HRP-conjugated Goat Anti-Rabbit IgG(H + L) (1:5000, #SA00001-2, Proteintech, Wuhan, China), HRP-conjugated Goat Anti-Mouse IgG(H + L) (1:5000, #SA00001-1, Proteintech, Wuhan, China).

### 2.14. Co-Immunoprecipitation (CO-IP)

The protein lysate was divided into three portions: one as the input and the other two were supplemented with IgG (B900620, Proteintech, Wuhan, China) and the primary antibodies, respectively. After incubating overnight, magnetic beads were added to each portion and incubated overnight at 4 °C again. The next day, the lysate was mixed with loading buffer and boiled at 100 °C for 5 min. We then followed the steps as described for Western blot.

### 2.15. Immunofluorescence (IF) Staining

Cells with different treatments were fixed with 4% paraformaldehyde and penetrated with 0.5%TritonX-100 (IT9100, Solarbio, Beijing, China). After blocking with 5%BSA for 1 h at room temperature, the cells were incubated with primary antibodies overnight at 4 °C. The next day, cells were incubated with secondary antibodies and stained with DAPI (S2110, Solarbio, Beijing, China). Then the samples were observed by a fluorescence microscope (ZEISS, Oberkochen, Germany). The primary antibodies were as follows: anti-AIM2 (1:200, #PH11499, Abmart, Shanghai, China), anti-caspase-3 (1:200, #TA6311, Abmart, Shanghai, China), anti-GSDME (1:200, #P79886, Abmart, Shanghai, China), anti-G9a (1:2000, #66689-1-Ig, Proteintech, Wuhan, China), anti-RIG-I (1:400, #20566-1-AP, Proteintech, Wuhan, China), anti-STAT1 (1:400, #10144-2-AP, Proteintech, Wuhan, China). The secondary antibodies were as follows: CoraLite594-conjugated Goat Anti-Rabbit IgG(H + L) (1:500, #SA00013-4, Proteintech, Wuhan, China), CoraLite488-conjugated Goat Anti-Mouse IgG(H + L) (1:500, #SA00013-1, Proteintech, Wuhan, China).

### 2.16. Statistical Analysis

All data are presented as the mean ± standard deviation (SD) from three independent experiments. A normality test was performed on the data distribution. Student’s *t*-test and one-way ANOVA were performed to compare two or more groups, *p* values less than 0.05 were regarded as statistically significant. GraphPad Prism software 8.0 was used to analyze data.

## 3. Results

### 3.1. Knockdown of G9a and Its Inhibitor Suppress Cells Proliferation

**Figure 1:**
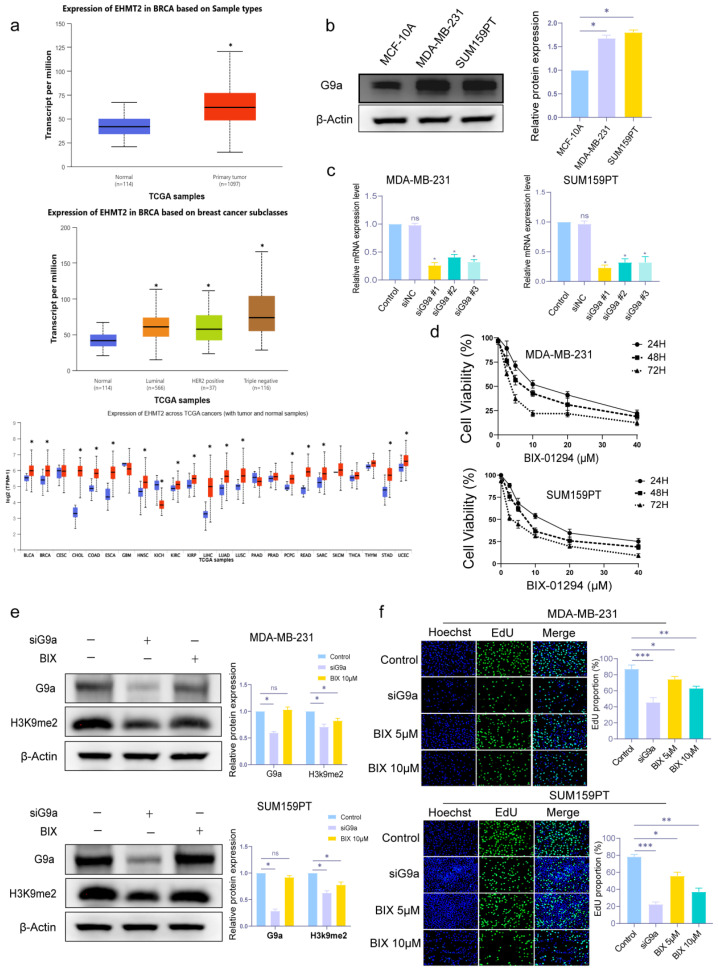
Knockdown of G9a suppresses cell proliferation. (**a**) Comparison of G9a levels between normal and tumor samples in the TCGA database. (**b**) Expression of G9a in normal breast cells and TNBC cells. (**c**) Knockdown efficiency of G9a in MDA-MB-231 and SUM159PT cells. (**d**) MDA-MB-231 and SUM159PT cells were treated with BIX-01294 (BIX) in specific concentrations (0, 5, 10, 20, 40 μmol/L) for 24, 48, and 72h. Cell viability was determined by CCK8 assay. (**e**) The influence of G9a knockdown and BIX on the expression levels of G9a and H3K9me2 in MDA-MB-231 and SUM159PT cells. (**f**) MDA-MB-231 and SUM159PT cells were treated with BIX (5 μmol/L and 10 μmol/L) for 24 h and stained with EdU. Hoechst means nuclear staining. Scale bar: 100 μm. * *p* < 0.05, ** *p* < 0.01, *** *p* < 0.001, ns, no significant. Original images of Western blotting can be found in Supplementary Materials.

G9a can catalyze H3K9me2 [15], and numerous investigations have shown that G9a is overexpressed in BC [18,19]. In Figure 1a, we detected G9a expression in BC and normal tissues from the TCGA database, and it showed that G9a expression is significantly up-regulated in breast cancer tissues compared to normal breast tissues, especially in TNBC. To validate this finding, we detected the levels of G9a by Western blot. Consistent with the TCGA data, G9a expression was higher in TNBC cell lines (MDA-MB-231 and SUM159PT) than in the normal breast epithelial cell line MCF-10A (Figure 1b). Then we used siRNAs to knock G9a down. Among all siRNAs, siG9a #1 showed the highest efficiency and was employed in the following experiments (Figure 1c). CCK8 assay showed that BIX-01294 (BIX), the inhibitor of G9a, could inhibit the proliferation of TNBC cells in a time- and dose-dependent manner (Figure 1d), and the IC50 values in MDA-MB-231 were 12.65 μmol/L, 7.69 μmol/L, and 3.62 μmol/L for 24 h, 48 h, and 72 h, while the corresponding values in SUM159PT were 11.84 μmol/L, 7.26 μmol/L, and 3.19 μmol/L.

We could also find that G9a knockdown can inhibit the expression of G9a and the methylation of H3K9me2, and BIX can suppress the methylation of H3K9me2 but has no significant effect on the expression of G9a (Figure 1e) (* *p* < 0.05). In EdU assay (Figure 1f), both G9a knockdown and BIX groups significantly reduced EdU proportion compared to the control group (* *p* < 0.05, ** *p* < 0.01, *** *p* < 0.001). These results showed that G9a knockdown and its inhibitor BIX suppressed cell proliferation, whereas BIX influenced the catalytic effect of G9a instead of its expression.

### 3.2. Knockdown and Inhibition of G9a Promote Apoptosis

**Figure 2:**
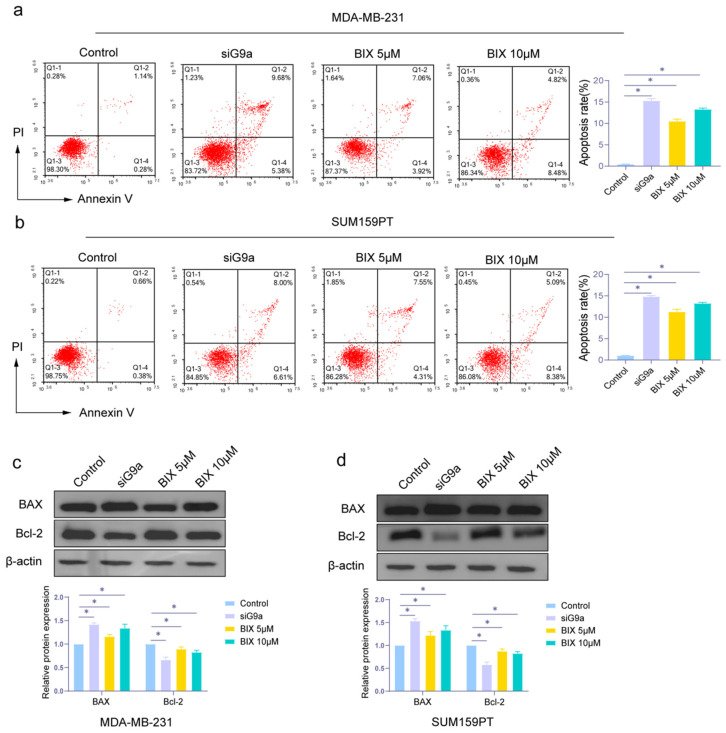
Knockdown of G9a induces apoptosis in MDA-MB-231 and SUM159PT cells. (**a**,**b**) Apoptosis rates in MDA-MB-231 and SUM159PT cells. (**c**,**d**) The expression levels of BAX and Bcl-2 in MDA-MB-231 and SUM159PT cells treated with or without si-G9a or BIX. * *p* < 0.05. Original images of Western blotting can be found in Supplementary Materials.

In order to investigate the influence of G9a in cell apoptosis, we used flow cytometric analysis to evaluate. As shown in Figure 2a,b, apoptosis rate was increased upon G9a knockdown in TNBC cells, and the pharmacological inhibition of G9a activity also induced apoptosis. The expression levels of BAX (a pro-apoptotic protein) and Bcl-2 (an anti-apoptotic protein) [33] were measured by Western blot assay. As can be seen in Figure 2c,d, BAX had higher expression in the knockdown group and the BIX group, compared with the control group. The results demonstrated that inhibition of G9a function could induce cell apoptosis in TNBC cells.

### 3.3. Knockdown and Inhibition of G9a Enhance the Suppression of Invasion and Migration in TNBC Cells

**Figure 3:**
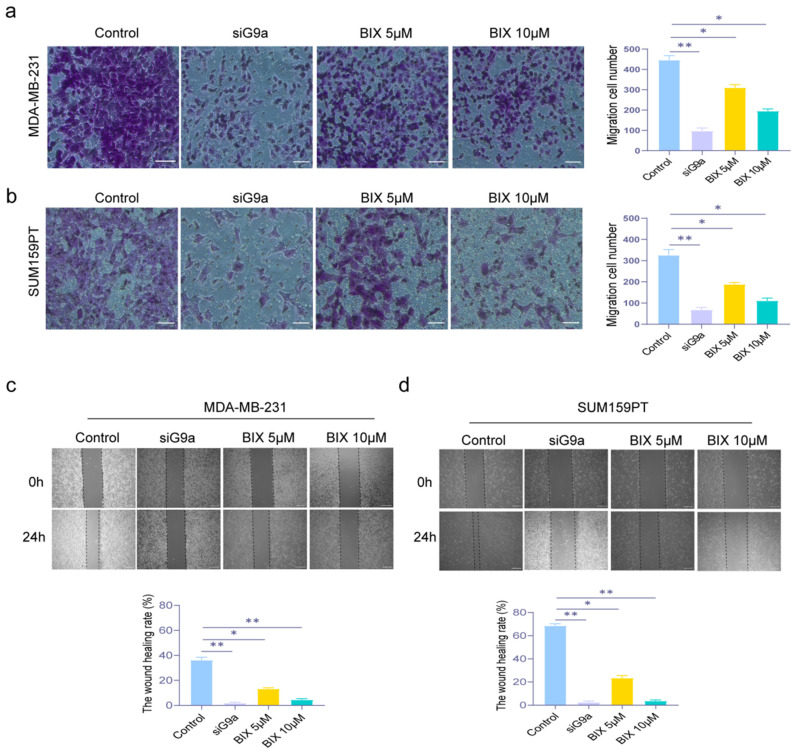
Knockdown of G9a inhibits invasion and migration ability in MDA-MB-231 and SUM159PT cells. (**a**,**b**) Transwell assay was used to measure the invasive ability of MDA-MB-231 and SUM159PT cells treated with or without si-G9a or BIX. Scale bar: 50 μm. (**c**,**d**) Wound healing assay examined the ability of migration in MDA-MB-231 and SUM159PT cells treated with or without si-G9a or BIX. Scale bar: 100 μm. * *p* < 0.05, ** *p* < 0.01.

The potential to metastasize is a crucial trait of malignant tumors, including TNBC, which is known for its high invasiveness and recurrence. To evaluate the two critical processes in metastasis—cell invasion and migration—we employed the Transwell (for invasion) and Wound Healing (for migration) assays. In Figure 3a,b, Both G9a knockdown and BIX groups significantly reduced the number of invasive cells compared to the control group. And there was the same trend in Figure 3c,d, that knockdown and inhibition of G9a resulted in lower wound healing rates relative to the control group (* *p* < 0.05, ** *p* < 0.01). The results indicated that G9a knockdown and BIX inhibited the invasion and migration abilities in TNBC cells.

### 3.4. G9a Affects Pyroptosis in TNBC Cells MDA-MB-231 and SUM159PT

**Figure 4:**
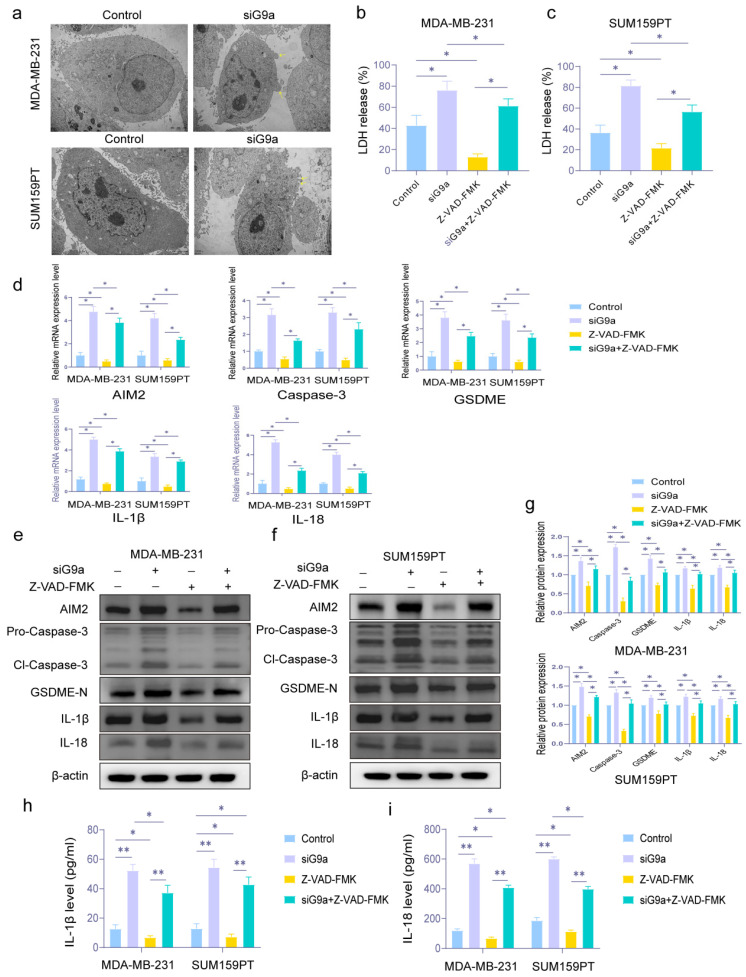
Knockdown of G9a induces pyroptosis in MDA-MB-231 and SUM159PT cells. (**a**) Morphologic features of MDA-MB-231 and SUM159PT cells on TEM. Cells exhibited membrane blebbing after G9a had been knocked down. Magnification, 10,000×, scale bar: 2 μm, TEM: Transmission electron microscopy. (**b**,**c**) The rate of LDH release in MDA-MB-231 and SUM159PT cells treated with or without si-G9a or Z-VAD-FMK. (**d**) The mRNA expression levels of AIM2, Cl-Caspase-3, GSDME-N, IL-1β, and IL-18 in MDA-MB-231 and SUM159PT cells treated with or without si-G9a or Z-VAD-FMK. (**e**,**f**) Western blot analysis of AIM2, Cl-Caspase-3, GSDME-N, IL-1β, and IL-18 expression in MDA-MB-231 and SUM159PT cells treated with or without si-G9a or Z-VAD-FMK. (**g**) Statistical graph of protein relative expression levels. (**h**,**i**) ELISA analysis for the detecting pyroptosis-associated inflammatory factors IL-1β and IL-18 in MDA-MB-231 and SUM159PT cells treated with or without si-G9a or Z-VAD-FMK. * *p* < 0.05, ** *p* < 0.01. Original images of Western blotting can be found in Supplementary Materials.

In terms of pyroptosis, we used TEM to observe the changes in cell membranes. It is generally recognized that the most obvious morphological alteration of pyroptosis is the swelling and blebbing of the cell membrane. As shown in Figure 4a, knockdown of G9a in MDA-MB-231 and SUM159PT exhibited membrane blebbing.

LDH is also released through membrane permeabilization during pyroptosis. In Figure 4b,c, when G9a was knocked down, the level of released LDH increased. We also used Z-VAD-FMK, known as an inhibitor of pyroptosis, for secondary verification. The Z-VAD-FMK group decreased LDH release and reduced the rise in LDH brought on by G9a knockdown (* *p* < 0.05).

Considering the major proteins of pyroptosis (AIM2, Cl-Caspase-3, GSDME-N, IL-18, and IL-1β), we estimated the mRNA level and protein expression by qRT-PCR and Western blot. In Figure 4d, we could see that the knockdown group had greater relative mRNA expression levels of genes related to pyroptosis. As above, the addition of the pyroptosis inhibitor reversed the pro-pyroptosis effect of G9a (* *p* < 0.05). The expression level of these proteins, shown in Figure 4e to g, also had the same performance as the result of qRT-PCR (* *p* < 0.05).

ELISA assay was used to test the levels of IL-18 and IL-1β. As shown in Figure 4h,i, the levels of IL-18 and IL-1β in TNBC cells were increased in the knockdown group. These trends were consistent with the Western blot results. (* *p* < 0.05, ** *p* < 0.01). Similarly, pyroptosis inhibitors had the potential to counteract this impact.

**Figure 5:**
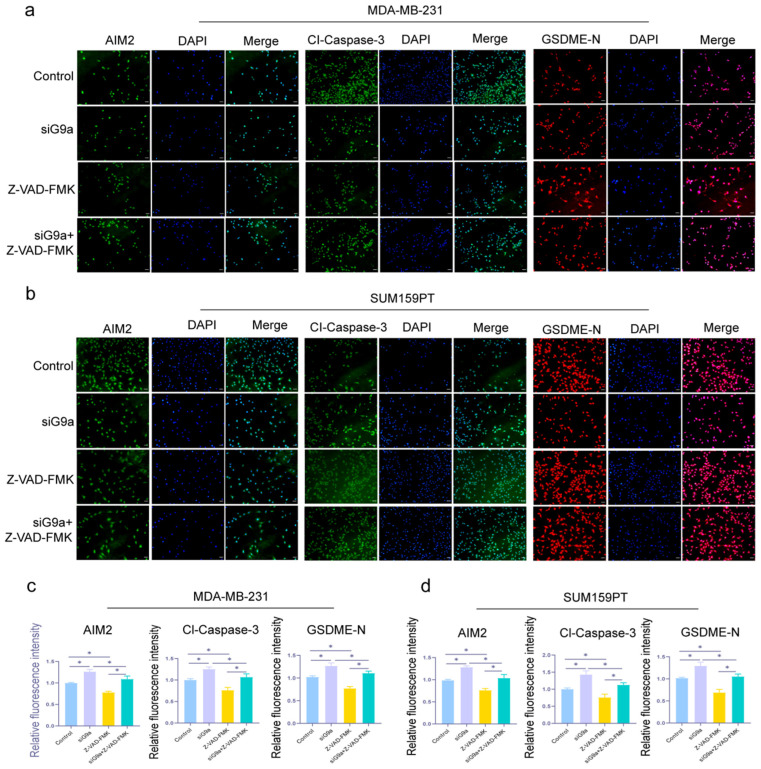
Knockdown of G9a promotes fluorescence intensity of pyroptosis-related proteins in MDA-MB-231 and SUM159PT cells. (**a**,**b**) The fluorescence intensity of MDA-MB-231 and SUM159PT cells treated with or without si-G9a or Z-VAD-FMK by IF staining. (**c**,**d**) Quantitative immunofluorescence analysis of AIM2, Cl-Caspase-3, and GSDME-N in MDA-MB-231 and SUM159PT cells. Scale bar: 100 μm. * *p* < 0.05.

We also examined the three proteins (AIM2, Cl-Caspase-3, and GSDME-N) by IF assay, and the results confirmed the tendencies shown in the previous experiments. In Figure 5, these proteins had higher fluorescence intensity in the knockdown group versus the control group (* *p* < 0.05). The results above suggested that G9a played a role in the process of pyroptosis, and pyroptosis was induced when the function of G9a had been suppressed in TNBC cells.

### 3.5. G9a Regulates Pyroptosis in TNBC Cells via RIG-I/STAT1/GSDME Signaling Pathway

**Figure 6:**
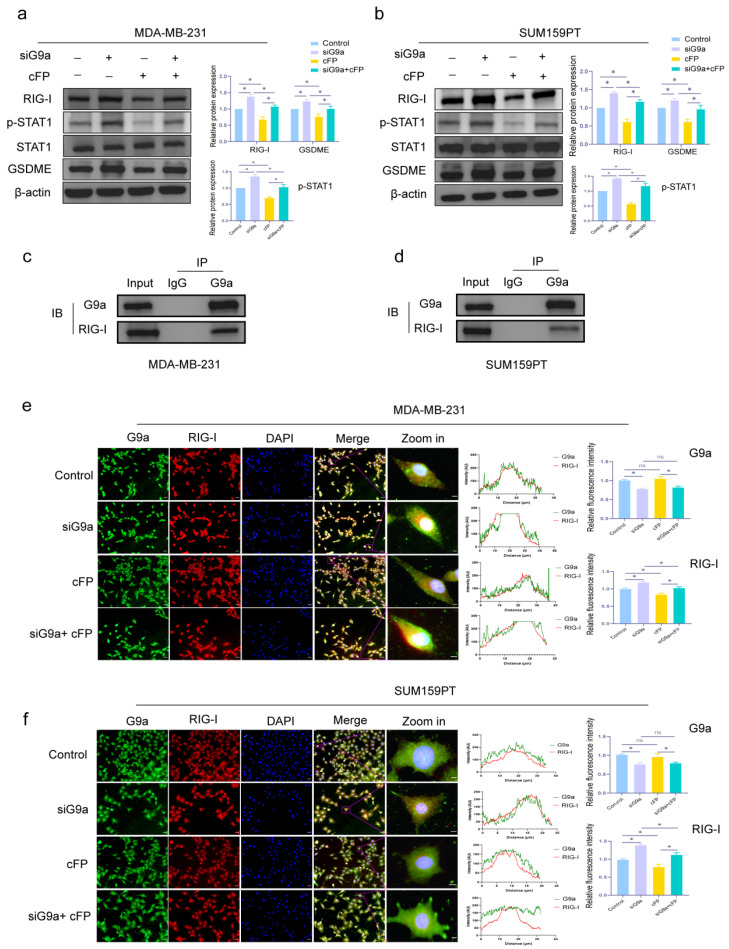
Knockdown of G9a promotes the expression of RIG-I in MDA-MB-231 and SUM159PT cells. (**a**,**b**) Western blot assay was used to evaluate the protein expression levels of RIG-I, p-STAT1, and GSDME in MDA-MB-231 and SUM159PT cells treated with or without si-G9a or cFP. (**c**,**d**) CO-IP assay was used to detect the interaction of G9a and RIG-I in MDA-MB-231 and SUM159PT cells. (**e**,**f**) Fluorescence intensity and co-localization of G9a and RIG-I in MDA-MB-231 and SUM159PT cells treated with or without si-G9a or cFP. Scale bar: 100 μm, cFP: Cyclo (Phe-Pro). * *p* < 0.05, ns, no significant. Original images of Western blotting can be found in Supplementary Materials.

RIG-I has been implicated in cancer development and progression [34,35,36], and also plays a role in pyroptosis [25,37]. As shown in Figure 6a,b, the expression of RIG-I, p-STAT1, and GSDME increased in G9a knockdown group, and decreased in the Cyclo (Phe-Pro) (cFP) group. Moreover, when cFP was added to the cells which had been knocked G9a down, the promoting effect of GSDME caused by inhibiting G9a was partially reversed (* *p* < 0.05).

To explore if G9a could interact with RIG-I, we performed CO-IP assay. In Figure 6c,d, the results showed that G9a and RIG-I might interact in MDA-MB-231 and SUM159PT. The IF assay in Figure 6e,f also revealed that the fluorescence intensity of RIG-I was higher in the knockdown group, and the promoting effect was diminished with the addition of cFP (* *p* < 0.05).

**Figure 7:**
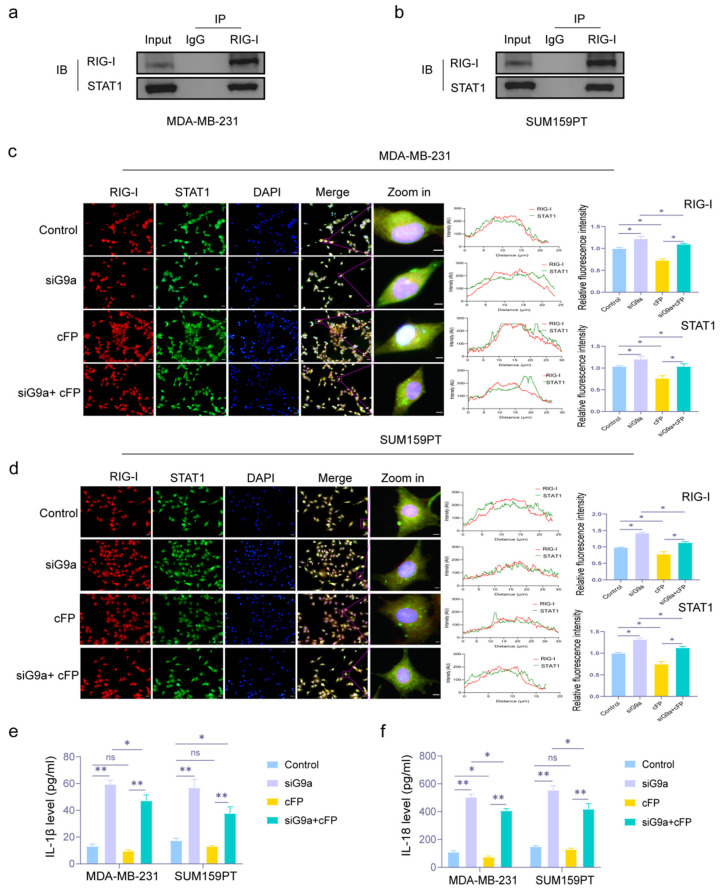
Knockdown of G9a activates the RIG-1/STAT1/GSDME pathway to motivate the pyroptosis process in MDA-MB-231 and SUM159PT cells. (**a**,**b**) CO-IP assay was used to determine the interaction of RIG-I and STAT1 in MDA-MB-231 and SUM159PT cells. (**c**,**d**) Fluorescence intensity and co-localization of RIG-I and STAT1 in MDA-MB-231 and SUM159PT cells treated with or without si-G9a or cFP. Scale bar: 100 μm. (**e**,**f**) ELISA was used to evaluate the expression levels of IL-1β and IL-18 in MDA-MB-231 and SUM159PT cells after treating with or without si-G9a or cFP. * *p* < 0.05, ** *p* < 0.01, ns, no significant. Original images of Western blotting can be found in Supplementary Materials.

Then we also tested whether RIG-I and STAT1 interacted. CO-IP assay showed that there was an interaction between RIG-I and STAT1 (Figure 7a,b). In Figure 7c,d, we also observed that G9a knockdown increased the fluorescence intensity and co-localization (yellow spot) of RIG-I and STAT1, suggesting their enhanced interaction. And cFP could partially reverse this effect (* *p* < 0.05). Then we estimated the expression of IL-1β and IL-18 by ELISA. In Figure 7e,f, the levels of IL-1β and IL-18 were increased in the siG9a group but reduced in the cFP group. cFP effectively reduced the up-regulation of the two pyroptosis-related inflammatory factors induced by G9a knockdown (* *p* < 0.05).

The above results revealed that G9a regulated pyroptosis in TNBC cells by RIG-I/STAT1/GSDME pathway.

## 4. Discussion

In this study, we revealed that inhibiting G9a suppresses the proliferation, decreases invasive and migratory capacities, and induces cell death, including apoptosis and pyroptosis in TNBC cells (MDA-MB-231 and SUM159PT). Moreover, we also discover that the RIG-I/STAT1/GSDME pathway may be responsible for the underlying mechanism of G9a involved in the pyroptosis process. We provide the first evidence elucidating how G9a regulates pyroptosis at the cellular level in TNBC cells, thereby revealing a novel mechanistic insight.

Due to a paucity of receptor indicators, TNBC has the worst prognosis among all subtypes of BC, and chemotherapy remains the important option for advanced TNBC [38]. Hence, identifying a therapeutic target is critical for patients with TNBC.

Studies demonstrated that G9a is overexpressed in various tumors and its exhaustion helps to alleviate the malignant manifestations of cancer [39,40,41,42]. In the present study, we found that the level of G9a expression in TNBC cells (MDA-MB-231 and SUM159PT) was higher than that in normal breast cells (MCF-10A). When G9a was knocked down, the abilities of proliferation, invasion, and migration of TNBC cells were reduced, while cell death was promoted. We also noticed that G9a knockdown reduced both G9a expression and H3K9me2 methylation levels, whereas the inhibitor (BIX) reduced H3K9me2 methylation but had no significant effect on G9a expression, which was consistent with the prior work [43].

Pyroptosis is a specific form of cell death characterized by cell swelling, membrane pore formation, and the release of pro-inflammatory cytokines that plays an important role in cancer. Three major pathways have been identified to activate pyroptosis: the canonical pathway mediated by Caspase-1, the non-canonical pathway directed by caspase-4/5/11, and the other pathways mediated by Caspase-3/GSDME and Caspase-8 [44]. GSDME can be cleaved by Caspase-3, and forms holes on cell membranes, causing the loss of membrane integrity and releasing the IL-1β and IL-18, which leads to cell death [45,46]. In BC, GSDME expression was reported downregulated [47]. Li et al. [48] found that Dihydroartemisinin could promote the expression of AIM2, caspase-3, and GSDME led pyroptosis in BC. In this study, we also found that G9a knockdown could stimulate the expression of the pyroptosis-related proteins, and TEM revealed changes in the cell membranes caused by pyroptosis. At the same time, we used an inhibitor of pyroptosis (Z-VAD-FMK), for secondary validation. We were surprised to find that the effects caused by knockdown and inhibitor of G9a could be partially reversed by Z-VAD-FMK. These results confirmed that suppressing the function of G9a could induce pyroptosis in TNBC cells.

RIG-I is a cytoplasmic pattern recognition receptor (PRR) that plays an important role in the innate immune response [26]. A meta-analysis showed that RIG-I was involved in the regulation of development and metastasis of BC [30], while Elion and co-authors found that RIG-I agonists could induce cell death in BC [31].

Signal transducer and activator of transcription 1 (STAT1) is a transcription factor involved in multiple intracellular signaling pathways [49]. STAT1 also plays an anti-cancer role in the progression of BC and pyroptosis [50]. BC patients with higher STAT1 activation had longer relapse-free and overall survival [51], and a longer survival period without distant metastasis [52]. While another study showed that STAT1 expression in BC cells was significantly lower than that in normal breast epithelial cells, and STAT1-deficient mice developed spontaneous breast tumors [53]. Various studies indicated that cytokines could induce pyroptosis by activating STAT1 [54,55]. Wang et al. also proved that STAT1 upregulated the inflammasome related to pyroptosis [56].

RIG-I induced cell death by activating STAT1 in hepatocellular carcinoma [57], and knockdown of RIG-I suppressed STAT1 expression and promoted radiotherapy resistance in Nasopharyngeal Carcinoma [58]. It also has been proven that RIG-I agonists activated STAT1 and NF-κB pathway in BC [31]. Interestingly, the efficacy of immunotherapy for hepatocellular carcinoma could be facilitated by promoting the STAT1/GSDME pyroptotic circuitry [59]. In the present study, we discovered that G9a, RIG-I, and STAT1 form an interaction network in TNBC cells. G9a knockdown activated the expression of RIG-I and p-STAT1 to promote GSDME. We also found that inhibiting the function of RIG-I suppressed the activation of p-STAT1 and GSDME. However, when an inhibitor of RIG-I (cFP) was given to the cells that had been knocked G9a down, the expression of RIG-I, p-STAT1, and GSDME were rebounded compared to the cFP group, suggesting that the G9a knockdown could partially reverse this decrease. Similarly, suppressing RIG-I function could also reverse the increase in pyroptosis-related inflammatory factors caused by G9a knockdown. These results suggested that RIG-I/STAT1/GSDME pathway might be the potential mechanism of G9a-induced pyroptosis in TNBC cells.

These findings would provide a theoretical basis for G9a being a therapeutic target, which would be of great significance for TNBC patients with poor treatment responses. However, there are still some limitations in this study. Firstly, the effects of G9a knockdown were exclusively investigated in TNBC cell lines in vitro; further studies in other BC subtypes and in vivo are needed to verify these results. Secondly, our findings indicated that there was an interaction between G9a and RIG-I; however, how G9a epigenetically regulates RIG-I warrants additional examination. Thirdly, as a pan-caspase inhibitor, Z-VAD-FMK could inhibit both pyroptosis and apoptosis. Consequently, the role of apoptosis in the effects of pyroptosis following the inhibitor treatment requires further investigation.

## 5. Conclusions

In conclusion, the study indicates that G9a is highly expressed in TNBC cells. Inhibiting G9a can suppress cell proliferation, invasion, and migration, and can induce cell apoptosis and pyroptosis. The mechanism by which G9a engages in the process of pyroptosis may be related to the RIG-I/STAT1/GSDME pathway. The findings demonstrate the therapeutic potential of inhibiting the function of G9a in TNBC cells.

## Data Availability

The original contributions presented in this study are included in the article/Supplementary Materials. Further inquiries can be directed to the corresponding authors.

## Supplementary Materials

The following supporting information can be downloaded at: https://www.mdpi.com/article/10.3390/biom16030345/s1, Western blots original figures.

## References

[1] Li Y., Zhang H., Merkher Y., Merkher Y., Chen L., Liu N., Leonov S., Chen Y. (2022). Recent advances in therapeutic strategies for triple-negative breast cancer. J. Hematol. Oncol..

[2] Harbeck N., Gnant M. (2017). Breast cancer. Lancet.

[3] Newport-Ratiu P.A., Hussein K.A., Carter T., Panjarian S., Jonnalagadda S.C., Pandey M.K. (2024). Unveiling the intricate dance: Obesity and tnbc connection examined. Life Sci..

[4] Ni Y., Schmidt K.R., Werner B.A., Koenig J.K., Guldner I.H., Schnepp P.M., Tan X., Jiang L., Host M., Sun L. (2019). Death effector domain-containing protein induces vulnerability to cell cycle inhibition in triple-negative breast cancer. Nat. Commun..

[5] Waks A.G., Winer E.P. (2019). Breast cancer treatment: A review. JAMA.

[6] Foulkes W.D., Smith I.E., Reis-Filho J.S. (2010). Triple-negative breast cancer. N. Engl. J. Med..

[7] Nagarajan K., Soundarapandian K., Thorne R.F., Li D., Li D. (2019). Activation of pyroptotic cell death pathways in cancer: An alternative therapeutic approach. Transl. Oncol..

[8] Thi H.T.H., Hong S. (2017). Inflammasome as a therapeutic target for cancer prevention and treatment. J. Cancer Prev..

[9] Ding J., Wang K., Liu W., She Y., Sun Q., Shi J., Sun H., Wang D.C., Shao F. (2016). Pore-forming activity and structural autoinhibition of the gasdermin family. Nature.

[10] Rogers C., Fernandes-Alnemri T., Mayes L., Alnemri D., Cingolani G., Alnemri E.S. (2017). Cleavage of dfna5 by caspase-3 during apoptosis mediates progression to secondary necrotic/pyroptotic cell death. Nat. Commun..

[11] Yan H., Luo B., Wu X., Guan F., Yu X., Zhao L., Ke X., Wu J., Yuan J. (2021). Cisplatin induces pyroptosis via activation of meg3/nlrp3/caspase-1/gsdmd pathway in triple-negative breast cancer. Int. J. Biol. Sci..

[12] Mahmoud Hewala T.I., El-Benhawy S.A., Elwany Y.N., Ellmasry H., Elrhawy A. (2024). Effect of radiotherapy on activating the pyroptotic cell death pathway in breast cancer patients: The role of serum gsdmd-ct, nlrp3 and il-18. Asian Pac. J. Cancer Prev..

[13] La Belle A., Khatib J., Schiemann W.P., Vinayak S. (2017). Role of platinum in early-stage triple-negative breast cancer. Curr. Treat. Options Oncol..

[14] Tan Y., Sun R., Liu L., Yang D., Xiang Q., Li L., Tang J., Qiu Z., Peng W., Wang Y. (2021). Tumor suppressor drd2 facilitates m1 macrophages and restricts nf-kappab signaling to trigger pyroptosis in breast cancer. Theranostics.

[15] Ni Y., Shi M., Liu L., Lin D., Zeng H., Ong C., Wang Y. (2024). G9a in cancer: Mechanisms, therapeutic advancements, and clinical implications. Cancers.

[16] Shinkai Y., Tachibana M. (2011). H3k9 methyltransferase g9a and the related molecule glp. Genes. Dev..

[17] Wang Y.F., Zhang J., Su Y., Shen Y.Y., Jiang D.X., Hou Y.Y., Geng M.Y., Ding J., Chen Y. (2017). G9a regulates breast cancer growth by modulating iron homeostasis through the repression of ferroxidase hephaestin. Nat. Commun..

[18] Casciello F., Al-Ejeh F., Kelly G., Brennan D.J., Ngiow S.F., Young A., Stoll T., Windloch K., Hill M.M., Smyth M.J. (2017). G9a drives hypoxia-mediated gene repression for breast cancer cell survival and tumorigenesis. Proc. Natl. Acad. Sci. USA.

[19] Mabe N.W., Garcia N.M.G., Wolery S.E., Newcomb R., Meingasner R.C., Vilona B.A., Lupo R., Lin C.C., Chi J.T., Alvarez J.V. (2020). G9a promotes breast cancer recurrence through repression of a pro-inflammatory program. Cell Rep..

[20] Liu X.R., Zhou L.H., Hu J.X., Liu L.M., Wan H.P., Zhang X.Q. (2018). Unc0638, a g9a inhibitor, suppresses epithelial-mesenchymal transition-mediated cellular migration and invasion in triple negative breast cancer. Mol. Med. Rep..

[21] Huo Z., Zhang S., Su G., Cai Y., Chen R., Jiang M., Yang D., Zhang S., Xiong Y., Zhang X. (2025). Immunohistochemical profiling of histone modification biomarkers identifies subtype-specific epigenetic signatures and potential drug targets in breast cancer. Int. J. Mol. Sci..

[22] Dong C., Wu Y., Yao J., Wang Y., Yu Y., Rychahou P.G., Evers B.M., Zhou B.P. (2012). G9a interacts with snail and is critical for snail-mediated e-cadherin repression in human breast cancer. J. Clin. Investig..

[23] Yoneyama M., Kikuchi M., Natsukawa T., Shinobu N., Imaizumi T., Miyagishi M., Taira K., Akira S., Fujita T. (2004). The rna helicase rig-i has an essential function in double-stranded rna-induced innate antiviral responses. Nat. Immunol..

[24] Franchi L., Eigenbrod T., Munoz-Planillo R., Ozkurede U., Kim Y.G., Arindam C., Gale M., Silverman R.H., Colonna M., Akira S. (2014). Cytosolic double-stranded rna activates the nlrp3 inflammasome via mavs-induced membrane permeabilization and k+ efflux. J. Immunol..

[25] Li Y., Guo M., Wang Q., Zhou H., Wu W., Lin H., Fan H. (2024). Glaesserella parasuis serotype 5 induces pyroptosis via the rig-i/mavs/nlrp3 pathway in swine tracheal epithelial cells. Vet. Microbiol..

[26] Mills E.L., Kelly B., O’Neill L.A.J. (2017). Mitochondria are the powerhouses of immunity. Nat. Immunol..

[27] Li Z., Zhou Y., Jia K., Yang Y., Zhang L., Wang S., Dong Y., Wang M., Li Y., Lu S. (2022). Jmjd4-demethylated rig-i prevents hepatic steatosis and carcinogenesis. J. Hematol. Oncol..

[28] Zhang L., Li Y., Zhou L., Zhou H., Ye L., Ou T., Hong H., Zheng S., Zhou Z., Wu K. (2023). The m6a reader ythdf2 promotes bladder cancer progression by suppressing rig-i-mediated immune response. Cancer Res..

[29] Song J., Zhao W., Zhang X., Tian W., Zhao X., Ma L., Cao Y., Yin Y., Zhang X., Deng X. (2022). Mutant rig-i enhances cancer-related inflammation through activation of circrig-i signaling. Nat. Commun..

[30] Li Y., Bai W., Zhang L. (2020). The overexpression of cd80 and isg15 are associated with the progression and metastasis of breast cancer by a meta-analysis integrating three microarray datasets. Pathol. Oncol. Res..

[31] Elion D.L., Jacobson M.E., Hicks D.J., Rahman B., Sanchez V., Gonzales-Ericsson P.I., Fedorova O., Pyle A.M., Wilson J.T., Cook R.S. (2018). Therapeutically active rig-i agonist induces immunogenic tumor cell killing in breast cancers. Cancer Res..

[32] Ghildiyal R., Sen E. (2017). Concerted action of histone methyltransferases g9a and prmt-1 regulates pgc-1alpha-rig-i axis in ifngamma treated glioma cells. Cytokine.

[33] Renault T.T., Dejean L.M., Manon S. (2017). A brewing understanding of the regulation of bax function by bcl-xl and bcl-2. Mech. Ageing Dev..

[34] Jiang Y., Zhang H., Wang J., Chen J., Guo Z., Liu Y., Hua H. (2023). Exploiting rig-i-like receptor pathway for cancer immunotherapy. J. Hematol. Oncol..

[35] Xu X.X., Wan H., Nie L., Shao T., Xiang L.X., Shao J.Z. (2018). Rig-i: A multifunctional protein beyond a pattern recognition receptor. Protein Cell.

[36] Zhao H., Zhang L., Du D., Mai L., Liu Y., Morigen M., Fan L. (2024). The rig-i-like receptor signaling pathway triggered by staphylococcus aureus promotes breast cancer metastasis. Int. Immunopharmacol..

[37] Li N., Chen J., Geng C., Wang X., Wang Y., Sun N., Wang P., Han L., Li Z., Fan H. (2022). Myoglobin promotes macrophage polarization to m1 type and pyroptosis via the rig-i/caspase1/gsdmd signaling pathway in cs-aki. Cell Death Discov..

[38] Vagia E., Mahalingam D., Cristofanilli M. (2020). The landscape of targeted therapies in tnbc. Cancers.

[39] Zhang L., Liang B., Xu H., Gong Y., Hu W., Jin Z., Wu X., Chen X., Li M., Shi L. (2022). Cinobufagin induces foxo1-regulated apoptosis, proliferation, migration, and invasion by inhibiting g9a in non-small-cell lung cancer a549 cells. J. Ethnopharmacol..

[40] Bergin C.J., Zouggar A., Haebe J.R., Masibag A.N., Desrochers F.M., Reilley S.Y., Agrawal G., Benoit Y.D. (2021). G9a controls pluripotent-like identity and tumor-initiating function in human colorectal cancer. Oncogene.

[41] Yin C., Ke X., Zhang R., Hou J., Dong Z., Wang F., Zhang K., Zhong X., Yang L., Cui H. (2019). G9a promotes cell proliferation and suppresses autophagy in gastric cancer by directly activating mtor. FASEB J..

[42] Liu S., Ye D., Guo W., Yu W., He Y., Hu J., Wang Y., Zhang L., Liao Y., Song H. (2015). G9a is essential for emt-mediated metastasis and maintenance of cancer stem cell-like characters in head and neck squamous cell carcinoma. Oncotarget.

[43] Kubicek S., O’Sullivan R.J., August E.M., Hickey E.R., Zhang Q., Teodoro M.L., Rea S., Mechtler K., Kowalski J.A., Homon C.A. (2007). Reversal of h3k9me2 by a small-molecule inhibitor for the g9a histone methyltransferase. Mol. Cell.

[44] Chen C., Ye Q., Wang L., Zhou J., Xiang A., Lin X., Guo J., Hu S., Rui T., Liu J. (2023). Targeting pyroptosis in breast cancer: Biological functions and therapeutic potentials on it. Cell Death Discov..

[45] Wu D., Chen Y., Sun Y., Gao Q., Yu B., Jiang X., Guo M. (2020). Gasdermin family: A promising therapeutic target for cancers and inflammation-driven diseases. J. Cell Commun. Signal..

[46] Rogers C., Erkes D.A., Nardone A., Aplin A.E., Fernandes-Alnemri T., Alnemri E.S. (2019). Gasdermin pores permeabilize mitochondria to augment caspase-3 activation during apoptosis and inflammasome activation. Nat. Commun..

[47] Kim M.S., Lebron C., Nagpal J.K., Chae Y.K., Chang X., Huang Y., Chuang T., Yamashita K., Trink B., Ratovitski E.A. (2008). Methylation of the dfna5 increases risk of lymph node metastasis in human breast cancer. Biochem. Biophys. Res. Commun..

[48] Li Y., Wang W., Li A., Huang W., Chen S., Han F., Wang L. (2021). Dihydroartemisinin induces pyroptosis by promoting the aim2/caspase-3/dfna5 axis in breast cancer cells. Chem. Biol. Interact..

[49] Wang W., Lopez McDonald M.C., Kim C., Ma M., Pan Z.T., Kaufmann C., Frank D.A. (2023). The complementary roles of stat3 and stat1 in cancer biology: Insights into tumor pathogenesis and therapeutic strategies. Front. Immunol..

[50] Mori H., Chen J.Q., Cardiff R.D., Penzvalto Z., Hubbard N.E., Schuetter L., Hovey R.C., Trott J.F., Borowsky A.D. (2017). Pathobiology of the 129:Stat1 (−/−) mouse model of human age-related er-positive breast cancer with an immune infiltrate-excluded phenotype. Breast Cancer Res..

[51] Widschwendter A., Tonko-Geymayer S., Welte T., Daxenbichler G., Marth C., Doppler W. (2002). Prognostic significance of signal transducer and activator of transcription 1 activation in breast cancer. Clin. Cancer Res..

[52] Yau C., Esserman L., Moore D.H., Waldman F., Sninsky J., Benz C.C. (2010). A multigene predictor of metastatic outcome in early stage hormone receptor-negative and triple-negative breast cancer. Breast Cancer Res..

[53] Chan S.R., Vermi W., Luo J., Lucini L., Rickert C., Fowler A.M., Lonardi S., Arthur C., Young L.J., Levy D.E. (2012). Stat1-deficient mice spontaneously develop estrogen receptor alpha-positive luminal mammary carcinomas. Breast Cancer Res..

[54] Yang X., Zuo X., Zeng H., Liao K., He D., Wang B., Yuan J. (2023). Ifn-gamma facilitates corneal epithelial cell pyroptosis through the jak2/stat1 pathway in dry eye. Investig. Ophthalmol. Vis. Sci..

[55] Wang S., Wu Z.Z., Zhu S.W., Wan S.C., Zhang M.J., Zhang B.X., Yang Q.C., Xiao Y., Li H., Mao L. (2023). Ctla-4 blockade induces tumor pyroptosis via cd8(+) t cells in head and neck squamous cell carcinoma. Mol. Ther..

[56] Wang J., Zhang F., Xu H., Yang H., Shao M., Xu S., Lyu F. (2022). Tlr4 aggravates microglial pyroptosis by promoting ddx3x-mediated nlrp3 inflammasome activation via jak2/stat1 pathway after spinal cord injury. Clin. Transl. Med..

[57] Hou J., Zhou Y., Zheng Y., Fan J., Zhou W., Ng I.O., Sun H., Qin L., Qiu S., Lee J.M. (2014). Hepatic rig-i predicts survival and interferon-alpha therapeutic response in hepatocellular carcinoma. Cancer Cell.

[58] Jing D., Zhou W., Shen L., Zhang Q., Xie W.T., Shen E., Li Z., Shen L.F., Sun L.Q. (2019). Rig-i promotes ifn/jak2 expression and the endoplasmic reticulum stress response to inhibit chemoradiation resistance in nasopharyngeal carcinoma. Cancer Med..

[59] Tu Y., Wu H., Zhong C., Liu Y., Xiong Z., Chen S., Wang J., Wong P.P., Yang W., Liang Z. (2025). Pharmacological activation of stat1-gsdme pyroptotic circuitry reinforces epigenetic immunotherapy for hepatocellular carcinoma. Gut.

