# Supplementary figures and images for: Targeting G9a Exerts Pleiotropic Suppression in Triple-Negative Breast Cancer Cells: Cooperatively Inducing Pyroptosis and Apoptosis
Source: Biomolecules. 2026 Feb 25;16(3):345. doi: 10.3390/biom16030345 (PMC13023577; doi:10.3390/biom16030345)

Western blots original figures

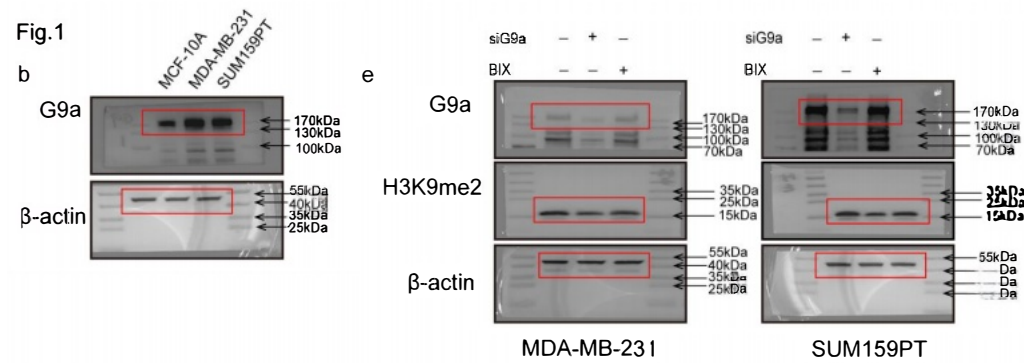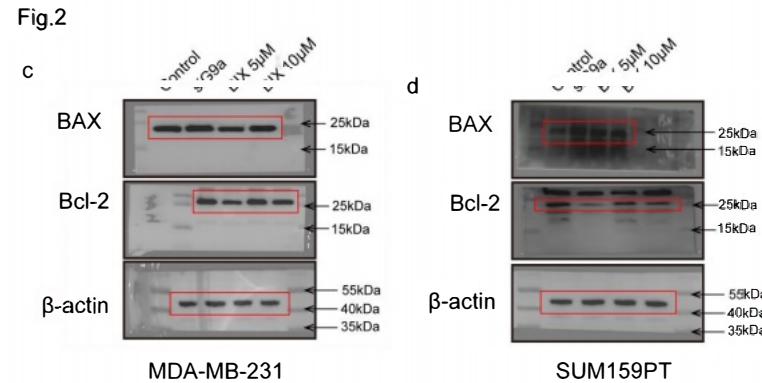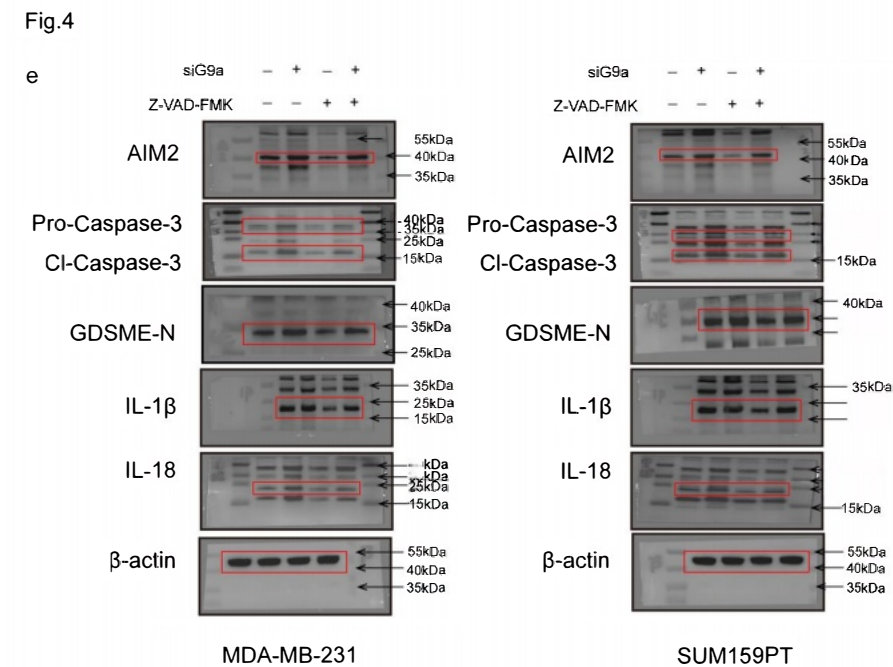

Fig.6

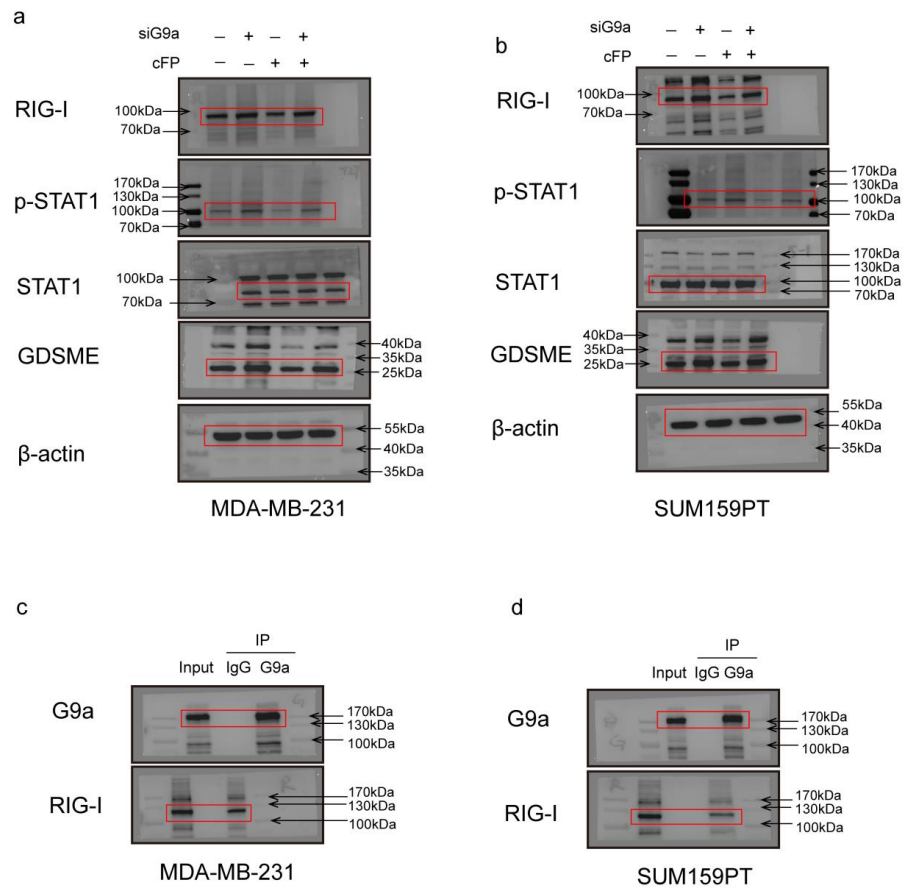

Fig.7

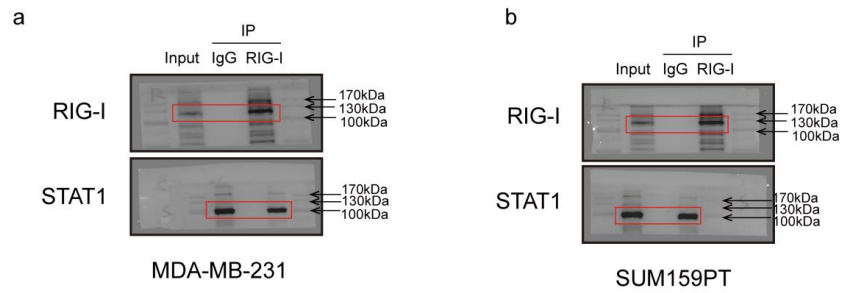

Supplement: Supplementary file 1 [file biomolecules-16-00345-s001.zip › biomolecules-4126249-supplementary.pdf]
